# Adjacent vessel sign and breast imaging reporting and data system are valuable for diagnosis of benign and malignant breast lesions

**DOI:** 10.1080/13102818.2014.974016

**Published:** 2014-10-29

**Authors:** Suhong Zhao, Ru Tan, Jianjun Xiu, Xianshun Yuan, Qingwei Liu

**Affiliations:** ^a^ Department of Radiology, The Second Hospital of Shandong University, Jinan City, Shandong Province, P.R. China; ^b^ Department of Radiology, Provincial Hospital, Shandong University, Jinan City, Shandong Province, P.R. China

**Keywords:** magnetic resonance imaging, breast cancer, adjacent vessel sign, breast imaging reporting and data system

## Abstract

The purpose of this study is to investigate whether an adjacent vessel sign (AVS) observed on the maximum intensity projections (MIPs) from the subtracted images can help distinguish between malignant and benign breast lesions and to test whether the combination of breast imaging reporting and data system (BI-RADS) category and AVS can increase the specificity and diagnostic accuracy of breast magnetic resonance imaging (MRI). The study included 63 histologically verified lesions which underwent dynamic breast MRI before biopsy. All magnetic resonance (MR) images were evaluated by two radiologists in consensus, who were unaware of the histopathological outcome. The MR images of all cases were analyzed according to BI-RADS-MRI assessment category. Levels of suspicion were reported as categories of I–V. The presence of vessels either entering the enhancing lesion or in contact with the lesion edge on MIP images was considered as the presence of AVS. Final analysis of 63 masses revealed 41 malignant lesions (65.1%) and 22 benign lesions (34.9%). Thirty seven out of 41 malignant lesions and 3 out of 22 benign lesions were associated with adjacent vessel, with highly significant difference between benign and malignant lesions (*P* < 0.001), especially for lesions smaller than 2.0 cm. The corresponding specificity, sensitivity and accuracy of contrast-enhanced 3.0-T breast were 86.4%, 82.9% and 84.1%, respectively. Based on BI-RADS-MRI category, the specificity, sensitivity and accuracy of breast MRI were 54.5%, 100% and 84.1%, respectively. After combining BI-RADS category and AVS, the specificity, sensitivity and accuracy of breast MRI were 90.9%, 82.9% and 85.7%, respectively. AVS can help differentiate malignant from benign breast lesions, especially for the lesions smaller than 2.0 cm. The combination of BI-RADS category and AVS can increase the specificity and the diagnostic accuracy of breast MRI.

## Introduction

Breast cancer is one of the most common cancers in women aged more than 40 years.[1] Early diagnosis of breast cancer has great importance for choosing appropriate clinical treatment and predicting prognoses. Magnetic resonance (MR) imaging (MRI) is one of the most important methods in the diagnosis of early breast cancer. The American College of Radiology Breast Imaging Reporting and Data System MRI (BI-RADS-MRI) lexicon was developed in order to standardize analyses and reporting of breast MR images.[2] It can predict the probability of malignancy for MR breast masses according to morphological and kinetic features,[3] but the overall specificity of breast MRI is relatively low.[4–6] In order to increase the specificity of breast MRI, several approaches have been proposed, such as diffusion-weighted imaging, MR spectroscopy and computer-aided diagnosis.[7–9] However, these approaches require additional scanning time and/or extra costs for hardware/software.

MR dynamic contrast-enhanced maximum intensity projection (MIP) imaging that can show tumor vessels and enhancing lesions clearly is based on MR imaging without additional sequences and scanning time.[10] In the last 10 years, researchers were trying to explore the value of vessel analysis in dynamic contrast-enhanced MRI for the diagnosis of whole-breast vascularity and the adjacent vessel sign (AVS) of a lesion or lesions.[11–20] Compared with whole-breast vascularity, AVS is more practical and generally applicable, especially on patients who have undergone mastectomy.[20] However, AVS is not enough to be used as a stand-alone diagnostic method, because its sensitivity and specificity for the diagnosis of breast cancer are only 74% and 89%, respectively.[20] AVS may be useful if combined with the BI-RADS-MRI lexicon. Ham et al. reported that in conjunction with the standard BI-RADS-MRI lexicon, AVS and increased ipsilateral whole-breast vasularity served as additional indicator for a poor prognosis.[21] In this study, we investigate whether the adjacent vessel is leading to an enhancing lesion observed on MIPs. The subtracted images can help distinguish between malignant and benign breast masses. In addition, we test whether the combination of BI-RADS-MRI and AVS can increase the specificity and diagnostic accuracy of breast cancer.

## Subjects and methods

### Patients

This retrospective study was approved by the local ethics committee. We reviewed 89 breast MR examinations performed at our hospital between May 2012 and October 2013, in which 26 cases were excluded due to chemotherapy or radiotherapy (*n* = 11), or lack of surgical confirmation (*n* = 15). The remaining 63 patients with an average age ± standard deviation of 43.78 ± 10.20 years were included in the analysis. After examination, all lesions were histologically verified by lumpectomy, mastectomy or biopsy at the Institute of Pathology in Provincial Hospital of Shandong University.

### MRI

All breast MR examinations were performed on a 3.0-T system (MagnetomVerio, Siemens, Germany) with an eight-channel dedicated breast coil. The patients were in the prone position, and both breasts were imaged simultaneously. Imaging was performed between day 7 and day 14 of the menstrual cycle for premenopausal women. The dynamic study was performed using an axial plane with a fat-saturated three-dimensional (3D) T1-weighted FLASH sequence. The parameters were: repetition time/echo time (TR/TE), 4.7/1.7; flip angle, 12^°^; field of view, 360 × 360 mm; matrix size, 384 × 296 and slice thickness, 1.2 mm. After unenhanced acquisition, 0.1 mmol/kg body weight of gadopentetatedimeglumine (GD-DTPA, Magnevist, USA) was applied intravenously at the rate of 2 ml/s followed by 10 ml saline flush. Dynamic contrast-enhanced image acquisition was started immediately after saline injection. The sequence was repeated nine times without time gaps. Each sequence lasted 59 seconds. For the generation of MR angiography, unenhanced images in the dynamic sequence were subtracted from the second series of contrast-enhanced images and MIP reconstruction was applied to the subtracted images.

All MR images were evaluated at a Siemens syngo workstation by two radiologists in consensus, who were blinded to the histopathologic outcome. Coronal and transverse MIPs were prepared from subtracted MR images and 3D rotation on the workstation for the assessment of AVS of breast lesions. The presence of vessels either entering the enhancing lesion or in contact with the lesion edge on MIP images was considered as the presence of AVS.[18–21]

The MR images of all cases were analyzed according to the BI-RADS-MRI assessment categories. According to morphology (lesion shape, margin and enhancement pattern) and enhancement kinetics (persistence, plateau or washout), lesions were divided into: category I, normal; category II, benign; category III, probably benign; category IV, suspicious malignancy and category V, highly suggestive of malignancy. According to the method previously reported by Schmitz et al.,[15] BI-RADS II lesions were classified as benign, namely negative diagnosis and were not combined with AVS. BI-RADS III–V lesions were classified as suspicious, namely positive diagnosis, and were combined with AVS. When the AVS was negative, BI-RADS III–V lesions were subsequently downstaged to negative diagnosis (BI-RADS II). If the AVS is positive, BI-RADS III–V lesions remained to be positive diagnosis.

### Statistical analysis

Possible associations between lesion sizes and the presence of the AVS were evaluated by *t*-test. The difference of the AVS between malignant and benign masses was compared by corrected Pearson's χ^2^ test and Fisher's exact probability test. The sensitivity, specificity, positive predictive value, negative predictive value and accuracy of the AVS for the presence of malignant masses were calculated. Statistical analysis was performed by SPSS version 17.0. *P* < 0.05 was considered statistically significant, and *P* < 0.001 was considered highly statistically significant.

## Results and discussion

### Malignant breast lesions are bigger than benign lesions in diameters

To investigate the sizes of breast lesions, *t*-test was used. In the 63 patients, our study revealed 41 malignant lesions including 36 invasive ductal carcinoma, 2 ductal carcinoma *in situ* (DCIS), 1 mucinous carcinoma, 1 comedo carcinoma and 1 invasive ductal carcinoma involving nerve, as well as 22 benign lesions including 7 atypical ductal hyperplasias, 2 adenosis, 2 mammary duct ectasias, 7 fibroadenomas, 1 hyperplasia with intraductal papilloma, 1 papilloma and 2 mastitis. The diameters of breast lesions ranged from 0.4 to 3.8 cm (mean size ± SD = 1.71 ± 0.68 cm). The mean size of malignant lesions (1.89 ± 0.57 cm) was statistically significantly higher than that of benign lesions (1.38 ± 0.76 cm) (*t* = 3.04, *P* < 0.05). These data indicated that malignant breast lesions were bigger than benign lesions in diameters.

### AVS can help distinguish between malignant and benign breast lesions, especially for the lesions smaller than 2.0 cm

**Figure 1.**
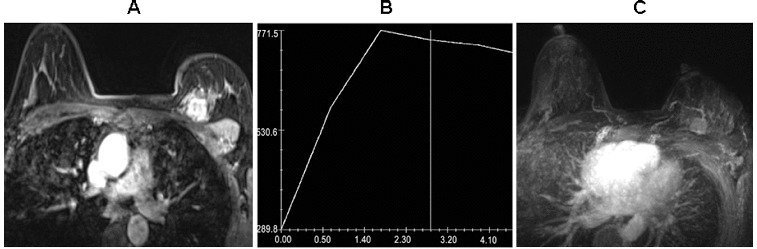
Invasive ductal carcinoma obtained from a 33-year-old woman. (A) Axial fat-suppressed contrast-enhanced axial image showing a 2.0 × 1.6 cm lobulated mass with heterogeneous enhancement in the upper-inner quadrant of the left breast. (B) Time–signal intensity curve of the mass shows a type III (washout) time curve. The *x*-axis shows the dynamic imaging beginning time in seconds, and the *y*-axis shows the intensity in arbitrary units. The mass was classified as a BI-RADS category IV lesion. (C) Maximum intensity projection reconstruction obtained from 3D T1-weighted subtraction image showing an adjacent vessel entering the mass.

**Figure 2.**
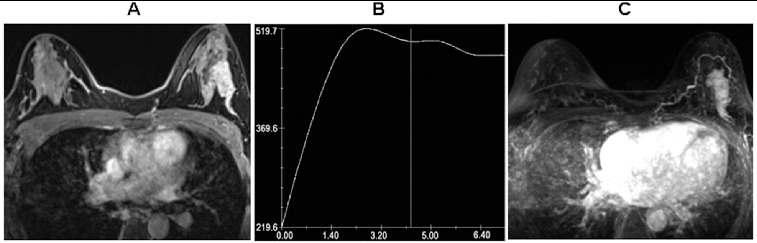
Hyperplasia with intraductal papilloma from a 27-year-old woman. (A) Axial fat-suppressed contrast-enhanced image showing a 2.2 × 2.9 cm irregular mass with heterogeneous enhancement in the upper-outer quadrant of the left breast. (B) Time–signal intensity curve of the mass shows a type II (plateau) time curve. The *x*-axis shows the dynamic imaging beginning time in seconds, and the *y*-axis shows the intensity in arbitrary units. The mass was classified as a BI-RADS category IV lesion. (C) Maximum intensity projection reconstruction obtained from 3D T1-weighted subtraction image showing an adjacent vessel in contact with the edge of the mass.

To identify the correlation between AVS and benign/malignant masses, *t*-test, corrected Pearson's χ^2^ test and Fisher's exact probability test were used. In the 63 lesions, 37 cases (58.7%) were associated with AVS, including 34 (91.9%) malignant lesions and 3 (8.1%) benign lesions (Table 1). Highly significant difference between benign and malignant lesions (χ^2^ = 25.57, *P* < 0.001) was observed. In addition, the corresponding specificity, sensitivity, positive predictive value, negative predictive value and the accuracy of contrast-enhanced 3.0-T breast were 86.4%, 82.9%, 91.9%, 73.1% and 84.1%, respectively (Table 1). For the 37 breast masses associated with adjacent vessel, the mean size of malignant lesions was 1.94 ± 0.58 cm and that of benign lesions was 2.90 ± 0.90 cm. There was statistically significant difference between them (*t* = −2.66, *P* < 0.05), suggesting that peripheral vascular syndrome appeared when the size of benign lesion was large. In the 21 breast lesions ≥ 2.0 cm, 19 were associated with AVS, including 16 malignant lesions and 3 benign lesions (Table 2). There was no significant difference between the two groups according to Fisher's exact test. In the 42 breast lesions < 2.0 cm, 18 malignant lesions were associated with the AVS and 5 malignant lesions and 19 benign lesions were not associated with AVS (Table 2). There was significant difference between malignant and benign lesions < 2.0 cm (*P* < 0.001) according to Fisher's exact (Figures 1 and 2). These data suggested that AVS could help distinguish between malignant and benign breast lesions, especially for the lesions smaller than 2.0 cm.

**Table 1.** Adjacent vessel sign of malignant and benign lesions on contrast-enhanced breast MRI.

Test result	Malignant	Benign	Total
Positive MRI	34 (TP)	3 (FP)	37
Negative MRI	7 (FN)	19 (TN)	26
Total	41	22	63
			
Parameter	Formula	*N*	Value
Sensitivity	TP/(TP + FN)	34/(34 + 7)	0.829
Specificity	TN/(TN + FP)	19/(19 + 3)	0.864
PPV	TP/(TP + FP)	34/(34 + 3)	0.919
NPV	TN/(TN + FN)	19/(19 + 7)	0.731
Accuracy	(TP + TN)/total	(34 + 19)/63	0.841

Note: FN, false negative; FP, false positive; TN, true negative; TP, true positive; PPV, positive predictive value; NPV, negative predictive value.

**Table 2.** Adjacent vessel sign in malignant and benign lesions in correlation with lesion size.

Subgroup	*N*	AVS (+)	AVS (−)	Total
≥2.0 cm	21			
Malignant		16	2	18
Benign		3	0	3
<2.0 cm	42			
Malignant		18	5	23
Benign		0	19	19

### The combination of BI-RADS-MRI category and AVS increased the specificity and the diagnostic accuracy of breast cancer

To test whether the combination of BI-RADS-MRI category and AVS can increase the specificity and diagnostic accuracy of breast cancer, the sensitivity, specificity, positive predictive value, negative predictive value and accuracy of malignant masses were calculated. According to BI-RADS criteria, among the 63 lesions, 12 cases (19%) were classified as BI-RADS category II, 6 cases (10%) were classified as BI-RADS category III, 29 cases (46%) were classified as BI-RADS category IV and 16 cases (25%) were classified as BI-RADS category V. Because there was no malignant lesion in BI-RADS category II, a sensitivity of 100% of contrast-enhanced 3.0-T breast was achieved. Among the 22 benign masses, 12 cases were negative, so the specificity of contrast-enhanced 3.0-T breast was 54.5% (Table 3). The positive predictive value, the negative predictive value and the accuracy of contrast-enhanced 3.0-T breast were 80.4%, 100% and 84.1%, respectively (Table 3). When the AVS was negative, four cases of BI-RADS III, eight cases of BI-RADS IV and three cases of BI-RADS V were downstaged (Table 4). Histopathological investigation revealed seven malignant lesions and eight benign lesions, which increased the specificity to 90.9% (20/22) and the accuracy to 85.7% (54/63). The sensitivity, positive predictive value and negative predictive value of contrast-enhanced 3.0-T breast were 82.9%, 94.4% and 74.1%, respectively (Table 5). These data suggested that the combination of BI-RADS-MRI category and AVS increased the specificity and the diagnostic accuracy of breast cancer.

**Table 3.** BI-RADS category of malignant and benign lesions.

Test result	Malignant	Benign	Total
Positive MRI	41 (TP)	10 (FP)	51
Negative MRI	0 (FN)	12 (TN)	12
Total	41	22	63
			
Parameter	Formula	*N*	Value
Sensitivity	TP/(TP + FN)	41/(41 + 0)	1.000
Specificity	TN/(TN + FP)	12/(12 + 10)	0.545
PPV	TP/(TP + FP)	41/(41 + 10)	0.804
NPV	TN/(TN + FN)	12/(12 + 0)	1.000
Accuracy	(TP + TN)/total	(41 + 12)/63	0.841

Note: FN, false negative; FP, false positive; TN, true negative; TP, true positive; PPV, positive predictive value; NPV, negative predictive value. Distribution of FP, TP and TN results was based on pathologic diagnosis and MRI categories. BI-RADS III–V lesions were positive MRI, and BI-RADS II lesions were negative MRI.

**Table 4.** BI-RADS category and adjacent vessel sign of lesions.

No. of cases	AVS (−)	AVS (+)	Total
BI-RADS II	11	1	12
BI-RADS III	4	2	6
BI-RADS IV	8	21	29
BI-RADS V	3	13	16
Total	26	37	63

**Table 5.** BI-RADS category of malignant and benign lesions according to adjacent vessel sign.

Test result	Malignant	Benign	Total
Positive MRI	34 (TP)	7 (FP)	41
Negative MRI	2 (FN)	20 (TN)	12
Total	36	27	63
			
Parameter	Formula	*N*	Value
Sensitivity	TP/(TP + FN)	34/(34 + 7)	0.829
Specificity	TN/(TN + FP)	20/(20 + 2)	0.909
PPV	TP/(TP + FP)	34/(34 + 2)	0.944
NPV	TN/(TN + FN)	20/(20 + 7)	0.741
Accuracy	(TP + TN)/total	(34 + 20)/63	0.857

Note: FN, indicates false negative; FP, false positive; TN, true negative; TP, true positive; PPV, positive predictive value; NPV, negative predictive value.

### Comparative analysis

In this study, we found that AVS is significantly more often observed in malignant breast lesions than in benign breast lesions (*P* < 0.001). Possible explanations are neoangiogenesis stimulated by breast malignant lesions, reduced flow resistance in tumor vessels and high tumor metabolism.[10] Our results are consistent with prior researches.[18,19] In our study, five invasive ductal carcinomas and two DCIS did not show adjacent vessel. The latter may be related to DCIS lesions exhibiting a benign blood flow pattern.[22] AVS was also seen in a minority of benign masses in our study, including two mastitis and one hyperplasia with intraductal papilloma. The reason may be the increasing metabolic demand of growing benign masses, which can also increase the blood supply.[18]

Like most previous studies, our study was focused on the relationship between lesion size and AVS.[18, 19] Fischer et al. observed that adjacent vessel in lesions smaller than or equal to 10 mm occurred significantly more often in benign lesions (*P* < 0.001). Dietzel et al. reported that AVS could be visualized significantly more often in malignant tumors exceeding 2 cm in size. In our study, the mean size of malignant lesions with adjacent vessel was 1.94 ± 0.58 cm and that of benign lesions was 2.90 ± 0.90 cm. There was no statistically significant difference between them (*P* > 0.05). We also observed that adjacent vessel in lesions smaller than 2.0 cm occurred significantly more often in malignant than in benign lesions (*P* < 0.001), but there was no statistically significant difference for lesions larger than or equal to 2.0 cm. Therefore, AVS can help distinguish between malignant and benign breast lesions, especially for lesions smaller than 2.0 cm. This finding also suggested that breast cancer is a hypervascular tumor. In our study, there was no significant difference between malignant masses with adjacent vessel (mean tumor size 1.94 cm) and malignant lesions without adjacent vessel (mean tumor size 1.67 cm) (*P* > 0.05). However, these findings must be supported by further studies with more samples.

In recent years, BI-RADS-MRI category has been widely used in the diagnosis of benign and malignant lesions according to morphological and kinetic features. It can predict the probability of malignancy for MR breast lesions.[3] An irregularly shaped mass with an irregular or speculated margin, heterogeneous enhancement (rim enhancement) with a fast initial enhancement and a washout pattern on the time–intensity curve may be malignant. Morphological and kinetic features suggestive of benignity are regularly shaped mass with a smooth margin, no enhancement or homogeneous enhancement and no washout pattern the on time–intensity curve.[23] However, the overall specificity of breast MRI is lower because some signs can be seen both in malignant and benign masses. A plateau curve can also be observed in invasive carcinomas.[23] Rim enhancement or subsequent peripheral washout is highly specific for the diagnosis of cancer (100%), but is less sensitive (51%).[24] Our results indicated that the sensitivity and specificity of contrast-enhanced 3.0-T breast were 100% and 54.5%, respectively, according to BI-RADS category. In order to improve the specificity of breast MRI, we combined the BI-RADS category with AVS.

When AVS was used with previous breast MRI (BI-RADS III–V) and patients without AVS were subsequently downstaged to negative breast MRI, four BI-RADS III cases, eight BI-RADS IV cases and three BI-RADS V cases were downstaged. Histopathology revealed seven malignant lesions (five invasive ductal carcinomas and two DCIS) and eight benign lesions, with the specificity and the diagnostic accuracy being increased to 90.9% (20/22 patients) and 85.7% (54/63 patients), respectively. These results showed that, in addition to morphological and kinetic features as defined in BI-RADS-MRI lexicon, AVS increased the specificity and the diagnostic accuracy of contrast-enhanced breast MRI.

However, this study has some limitations. First, the number of cases was small, and our results need to be validated with a large number of cases. Second, the evaluation of adjacent vessel and lesion was subjective, which may lead to some bias. Third, most of malignant lesions are invasive ductal carcinomas, appearing as mass-like enhancement. Therefore, we were not able to evaluate the difference of adjacent vessel between invasive and noninvasive cancers.

In conclusion, AVS can help distinguish between malignant and benign breast lesions, especially for lesions smaller than 2.0 cm. The combination of BI-RADS-MRI category and AVS can increase the specificity and the diagnostic accuracy of breast cancer.
